# The accuracy of 10 g monofilament use for clinical screening of diabetes peripheral neuropathy among Indian population

**DOI:** 10.1371/journal.pone.0297110

**Published:** 2024-02-23

**Authors:** Animesh Hazari, Vinaytosh Mishra, Praveen Kumar, Arun Maiya

**Affiliations:** 1 Gulf Medical University, Ajman, United Arab Emirates; 2 Manipal Academy of Higher Education, Manipal, Karnataka, India; Weill Cornell Medicine-Qatar, QATAR

## Abstract

**Objective:**

The purpose of this study was to test the diagnostic accuracy of the 10g monofilament to screen for diabetic peripheral neuropathy (DPN) in India. The study further assessed the effect of physical activity, footwear use, and occupation on the outcome.

**Methods:**

Non-probabilistic purposive sampling was used to recruit patients with T2DM to assess the diagnostic utility of the 10 g monofilament. 160 participants were recruited divided into 4 groups. Each group consisted of 40 participants with 20 under each category described as “Physical Worker Vs Non- physical worker” (n = 40), “Barefoot Vs Footwear” (n = 40), “Use of Slipper at Home Vs No-slippers use at home” (n = 40), “Agriculture Vs Non- agriculture” (n = 40). 10 g monofilament was used to detect the presence of protective sensation towards screening of DPN against biothesiometer (Vibration Pressure Threshold).

**Results:**

The area under the ROC (receiver operating characteristic) curve was 0.6 for identifying DPN using the 10 g monofilament. Physical work (p = 0.04), footwear (p = 0.04), slipper use at home (p = 0.02) and occupation (p = 0.02) impacted on the diagnostic utility of the 10g monofilament.

**Conclusions:**

This study shows that the 10 g monofilament has limited accuracy for detecting DPN in the Indian population and this is further affected by occupation, socioeconomic and religious practice.

## Introduction

Diabetes Mellitus is a global epidemic with a significantly high prevalence among the Indian population [1]. It is well known that diabetes mellitus leads to various complications amongst which diabetes peripheral neuropathy (DPN) is the most serious [2]. Studies report that around 50 percent of diabetes patients have the risk of developing DPN [3]. A previous study has reported a prevalence rate of 18–61% for DPN among the diabetic population in India which is a major factor in the etiopathogenesis of diabetic foot syndrome and ulceration [4]. It has been also reported that DPN imposes a significant economic burden on the diabetic sufferers due to its debilitating complications leading to a poor Quality of life in all domains [5, 6]. DPN causes serious nerve dysfunction leading to altered sensation and loss of protective sense eventually. Since the distribution is more peripheral, foot injuries and complications are at higher risk due to imposed plantar pressure in absence of intact sensory feedback underlying the disease. Thus, early screening becomes of utmost value. There are multiple qualitative and quantitative DPN screening tools available for clinical and research purposes. The gold standard tool used for DPN is Nerve Conduction Studies (NCV) [7]. However, being very costly, the use of NCV is limited for general practice at most Indian health setups. Thus, NCV doesn’t stand as a choice of DPN tool in community health. The widely used clinical tools in community include screening questionnaires that can indicate the possibility of DPN with considerable reliability and sensitivity. A commonly used tool is Michigan Neuropathy Screening Instrument (MNSI) which consists of two parts. Part ‘A’ consists of a self-administered questionnaire to understand the history and symptoms and part ‘B’ consists of physical examination including assessment of protective senses (using 10 g monofilament) and vibration (using Biothesiometer). It is economic and easy to use for clinical practice but could possess subjective errors. Although these questionnaires may take more time and lack precision against an objective measure, they are suitable for clinical diagnosis. In comparison to NCV, the most reliable and sensitive non-invasive objective instrument currently available for DPN is a Vibration Pressure Threshold device or Biothesiometer that yields quantified results which can also help to stratify the severity of DPN [8]. In addition, the Weinstein 10 g monofilament is widely used at major community healthcare settings in India as it is cost-effective and easy to use for clinical assessment of DPN screening in conjunction to other tools like MNSI. The reliability and validity of the 10 g monofilament have been tested against the NCV for diabetic population with DPN [9]. Various studies have reported the effectiveness of monofilament testing in screening DPN. It has been reported as very efficient for clinical use. The Weinstein monofilament exerts a pressure of 10 g which should be detected by any normal person to rule out the presence of DPN and it has been used to date with high success. However, there could be certain concerns for its sensitivity due to differences in demographic, social, ethnic, religious, and occupational characteristics of the diabetic person in a given geographical area. No studies report the findings on the sensitivity and specificity of the 10 g monofilament testing for DPN in geographical variation. As per our clinical experience, we understand that the accuracy of the 10 g monofilament for DPN use may be questionable due to various factors that influence lifestyle in a country like India. For instance, there could be variations in the thickness of the plantar fascia, variations in the use of footwear, personal care, occupations like long-standing, etc. In particular, the sociodemographic characteristics of the Indian population vary significantly which could have an impact on the 10 g monofilament accuracy for DPN and there is a gap in the literature suggesting no such findings. The research hypothesizes that 10g monofilament may not be accurate for clinical assessment of DPN among Indian population as suggested in MNSI ‘B’ which could be tested against more reliable biothesiometer for VPT. Thus, the study aims to reassess the accuracy of 10 g monofilament testing for DPN among the Indian population. The following are the objectives of the study:

1. To determine the accuracy of 10 g monofilament on the Indian Population over professional variation- Physical Worker Vs Non- physical worker
2. To determine the accuracy of 10 g monofilament on the Indian Population over economic variation- Barefoot Vs Footwear
3. To determine the accuracy of 10 g monofilament on the Indian Population over religious beliefs- Use of Slipper at Home Vs No use of slippers at home
4. To determine the accuracy of 10 g monofilament on the Indian Population over occupational variation–Agriculture Vs Non- agriculture

## Materials and methods

This section lists the method used in achieving research objectives stated in the earlier section.

### Study design and setting

The cross-sectional study was conducted at various community camps organized by the Diabetic Foot Clinic, Centre for Diabetes Care, Kasturba Hospital, Manipal, Karnataka, in India considering the selection of the sample population representation as per the stated objectives. To select a population with economic variation, the subjects were also recruited from a diabetes specialist tertiary care setting “Kasturba Medical College and Hospital, India”.

### Study population

A total of 160 types 2 diabetes participants (T2DM) were recruited in the study under the purposive sampling method. Participants were divided into 4 groups for testing the different hypotheses. Each group consisted of 40 participants with 20 under each category described as “Physical Worker Vs Non- physical worker” (n = 40), “Barefoot Vs Footwear” (n = 40), “Use of Slipper at Home Vs No-slippers use at home” (n = 40), “Agriculture Vs Non- agriculture” (n = 40). The following inclusion and exclusion criteria were defined for the proposed study.

1. Inclusion: Type 2 diabetes mellitus, duration of diabetes = more than 5 years, DPN with or without foot ulceration, with or without a history of amputation.
2. Exclusion: Peripheral Neuropathy other than diabetes origin such as Vitamin B12 deficiency, alcoholic neuropathy, polyneuropathy

### Study procedure

All participants were initially screened for diagnosis of T2DM based on their previous biochemistry investigations for blood sugar levels. Participants were then screened for inclusion and exclusion criteria after which they were given a written informed consent form to participate in the study. As part of Ph. D study, data was assessed in July 2023 from the main data repository available to the first author. Ethical approval was obtained for the study from the Institutional Ethics Committee, Kasturba Hospital. Participants were then grouped as per objectives 1–4 and then the accuracy of 10 g monofilament was tested for DPN, confirmed with presence of neuropathic symptoms (MNSI part A score ≥7, MNSI part B score ≥2) and abnormal VPT score on the biothesiometer device. On the VPT device, a score of more than 20 V was considered to have positive findings to confirm the presence of DPN [10]. The Semmes Weinstein 10 g monofilament testing was done as per the standard guidelines and the response was marked “Yes” or “No” based on the ability of the participant to perceive the 10 g pressure. The site of measurement was the great toe and heel for both tools. We first tested for the presence/absence of DPN using VPT and retested the same subject with 10 g monofilament. The findings of DPN screening using VPT were correlated with 10 g monofilament and reported as described below.

### Data analysis

To investigate 10g monofilament’s accuracy, the study used logistic regression as classification using VPT results as a dependents variable with results of 10 g monofilament (MFN) as the independent variable. The cutoff value of the probability used in the study was 0.5. The underlying hypothesis for the model is given below.

*H1*: *10g Monofilament is effective in the screening of Diabetic Peripheral Neuropathy among the Indian Population*

For further investigation of factors affecting the accuracy of the classification using 10 g monofilament, the study used the χ2-square test.

The underlying hypothesis for this objective is as follows:

*H2A*: *Accuracy of prediction of DPN using 10g Monofilament is independent of Physical work*.
*H2B*: *Accuracy of prediction of DPN using 10g Monofilament is independent of the Use of Footwear*
*H2C*: *Accuracy of prediction of DPN using 10g Monofilament is independent of the Use of Slippers at home*.
*H2D*: *Accuracy of prediction of DPN using 10g Monofilament is independent of Occupation (Agriculture)*

## Results

The important findings of the study have been presented in this section. Table 1 represents the demographic and clinical characteristics of all participants. Table 2 represents findings related to DPN for all participants. The findings on hypothesis testing are presented in Tables 3–5.

**Table 1:** Demographic and clinical characteristics of all participants.

Category of Participants (n = 160)	Age (Mean±SD)	BMI (Mean±SD)	Duration of Diabetes (Mean±SD)	HbA1c % (Mean±SD)
Physical Workers (n = 20)	49±10	23.9±4.1	7±3	7.2±1.1
Non-Physical Workers (n = 20)	54±6	26.7±5.3	6±4	7.9±1.5
Agriculture (n = 20)	51±7	25.3±4.7	8±2	7.3±2.2
Non-Agriculture (n = 20)	48±11	23.1±7.2	8±5	6.1±2.7
Barefoot (n = 20)	50±8	23.8±5.5	7±2	6.3±1.9
Footwear(n = 20)	52±5	24.1±3.9	9±3	7.9±1.7
Slippers at home (n = 20)	53±8	25.1±6.2	6±2	7.3±1.4
No slippers at home (n = 20)	55±4	24.5±5.1	9±5	6.5±1.6

**Table 2:** Findings related to confirmed DPN participants with altered VPT.

Category of Participants (n = 67)	MNSI ‘A’ (Mean±SD)	MNSI ‘B’ (Mean±SD)	VPT in Volts (Mean±SD)	Presence of Retinopathy	Presence of Nephropathy
Physical Workers (n = 10)	7±3	2±2	24±7	n = 3	n = 2
Non-Physical Workers (n = 7)	8±4	3±2	26±10	n = 5	n = 1
Agriculture (n = 10)	9±2	2±2	25±8	n = 2	n = 0
Non-Agriculture (n = 9)	7±5	2±2	31±17	n = 7	n = 4
Barefoot (n = 10)	8±2	2±2	28±5	n = 5	n = 1
Footwear(n = 5)	9±3	3±2	24±11	n = 2	n = 2
Slippers at home (n = 7)	8±2	3±2	25±9	n = 6	n = 0
No slippers at home (n = 9)	9±4	3±2	30±14	n = 5	n = 3

**Table 3:** Logistic regression model for classification.

	B	S.E.	Wald	df	Sig.
MFM	21.8	12118.6	.000	1	.999
Constant	-.585	.200	8.569	1	.003

**Table 4:** Contingency table for test of independence.

4(a): Physical Vs Non-Physical			
	Right	Wrong	Total
Physical	11	9	20
Non-Physical	15	5	20
4(b): Barefoot Vs Footwear			
Barefoot	11	9	20
Footwear	15	5	20
4(c): Slipper Vs No Slipper			
Slipper	15	5	20
No Slipper	10	10	20
4(d): Agriculture Vs Non-Agriculture			
Agriculture	10	10	20
Non-Agriculture	15	5	20

**Table 5:** Results for test of independence.

	χ2 Test	P-Value	Decision
H2A	4.37	0.04	Rejected
H2B	4.37	0.04	Rejected
H2C	5.24	0.02	Rejected
H2D	5.24	0.02	Rejected

**Fig 1:**
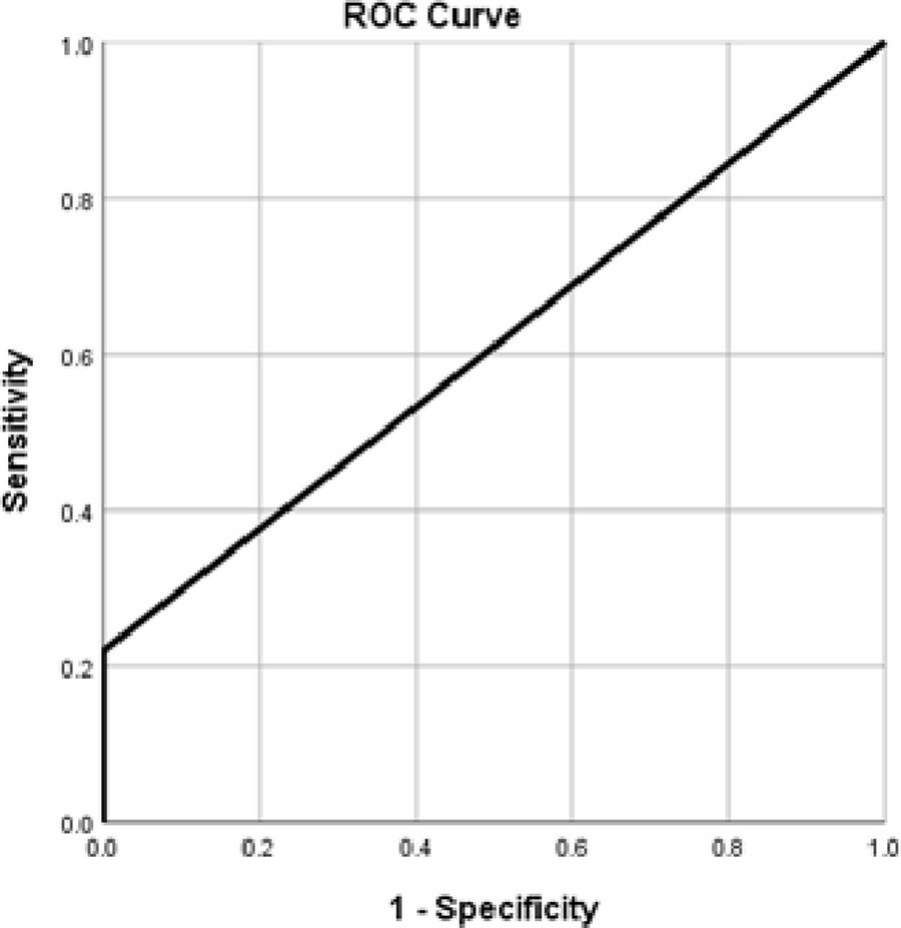
ROC curve for the classification.

For testing hypothesis 1 (H1), this study developed a logistic regression model as depicted in Table 3. The model suggests that 10 g monofilament is not a good classification tool for diabetic peripheral neuropathy among the given population. The ROC curve for the model is depicted in Fig 1.

For testing the rest of the hypothesis contingency table was created and listed in Table 4. The results of the hypothesis testing using the χ2 test are listed in Table 5.

## Discussion

In the present study, the aim was to find the accuracy of 10g monofilament testing for the screening of DPN in clinical practice among the Indian population. The study reported 160 Type 2 diabetes mellitus participants with demographic and clinical characteristics given in Tables 1 and 2 respectively. All participants were screened neuropathic symptoms using MNSI ‘A’. The VPT (Biothesiometer) was used to assess the vibration perception in all participants as part of physical examination in MNSI ‘B’ and confirm DPN above 20 V. Concurrently in the same visit, 10 g monofilament was used to screen the loss of protective sensation in all participants including confirmed cases of DPN with altered VPT (n = 67). Findings of the study suggested that use of 10 g Monofilament for DPN is limited among Indians with associated factors. Since the regression model was not found to be significant at a level of significance 0.05 (Table 3), it implies that unlike previous studies, 10 g monofilament may not be able to accurately be used to screen the diabetes peripheral neuropathy among Indian population in routine clinical examination. The finding was also supported by the plotted ROC curve (The area under the curve for the model is 0.6 only, Fig 1).

It was evident from the findings in Table 4, that monofilament testing resulted in a significant number of wrong predictions against the VPT. Out of 160 responses, 10 g monofilament resulted in 58 wrong responses (36%) suggesting its inaccuracy in clinical use for DPN screening among the Indian population. The findings of our study agreed with a previous study that reported the sensitivity and specificity of monofilament in screening DPN [11]. The study reported that the pooled sensitivity and specificity of monofilament testing for detecting DPN was 0.53 [11]. In contrast, a study compared 2g, 4g, and 10 g monofilament testing for screening DPN and found that the validity of 4g against 10 g was significant and could be used alternatively with a conclusion that 4 g monofilament is clinically useful for detecting DPN [12]. The findings of our study do not agree with it, and we found that when 10 g monofilament accuracy is questionable, the use of 4g could be of further limitation. However, we understand that there could be factors leading to such differential findings particularly due to variations in lifestyle, occupation, and socioeconomic differences in a given geographical area. Therefore, the study further investigated the factors affecting the accuracy of prediction. The contingency table for the hypotheses H2A, H2B, H2C, and H2D is given in Table 4. In a country like India, these differences prevail and the accuracy of 10 g monofilament for clinical use among the Indian population could be less. This hypothesis was supported by findings given in Table 5 which suggested that there was a significant difference for use of 10 g monofilament among the Indian population based on the physical and non-physical workers. Physical workers such as laborers have different working environments compared to non-physical workers like “white collars” which could lead to changes in their sensory adaptation to touch and pinprick response as tested by the 10 g monofilament. This finding was supported by the fact that compared to non-physical workers the number of wrong responses for a physical worker was more (9 compared to 5, Table 4). Thus, it could be suggested that the accuracy of 10 g monofilament is further compromised among the physical workers. The reason could be attributed to the difference in neuromuscular physiology between physical and non-physical workers (physically active vs sedentary comparison) [13]. Similar findings were seen for the difference in the socioeconomic condition of the participants who could afford footwear against barefoot. It was found that compared to footwear, there were more wrong responses to 10 g monofilament in barefoot Indian T2DM participants. The difference could be attributed to the use of footwear which could help to retain sensory protection more efficiently compared to the highly exposed and adapted loss of sensory input among the barefoot participants. The barefoot walking could lead to the increased displacement of the plantar fascia in presence of neuropathy [14] and thus could result in a negative finding of the 10 g monofilament. In addition, barefoot walking could also lead to changes in the arch of the foot and thickness of the plantar facia which could further add to difficulties in determining an intact sensory input using 10 g pressure via monofilament testing among the Indian population. We also understand that religious practice in India could have an impact on their lifestyle. For instance, Indians often do not use slippers at home, particularly Hindus, and thus the expected changes in the foot could be seen with barefoot walking. However, we are referring to the non-use of slippers in a protective environment compared to barefoot walking based on economic challenges. Nevertheless, we found that this factor also had a significant change in the findings of 10 g monofilament to detect DPN. There were 10 wrong responses (double) in participants without the use of slippers at home compared to 5 using slippers at home. The religious practice of the non-use of slippers at home could have affected the sensory system significantly leading to changes in the feet which could not be as accurately detected by 10 g monofilament. Studies have shown that footwear can enhance balance and control which is a part of the sensory feedback loop [15]. Lastly, we found that occupational background based on agriculture and non-agriculture could have significant changes in the sensory system among T2DM participants in India as we consider the majority of Indians from an agricultural background. Moreover, around 35% percent of the total Indian population do labor-intensive jobs. With this background, the use of 10g monofilament in the detection of diabetic peripheral neuropathy may not be a good approach as hinders the early detection of the disease. This finding was supported by a study conducted in Norway which concluded poor diagnostic utility of the 5.07/10 g Monofilament test for diabetes polyneuropathy, particularly among females and those with advanced DPN [16]. The limitation of 10 g monofilament for diagnosing DPN was further supported by previous researchers which didn’t recommend its use for polyneuropathy assessment and suggested that a comprehensive neurological examination should be done for early detection of DPN, against use of 10 g monofilament solely [17]. In our study, we included only active agricultural participants and do not have a background but are inactive currently. The findings between agriculture and non-agriculture T2DM participants suggested more inaccuracy of 10 g monofilament for the agriculture population in detecting DPN. The findings could be related to higher physical activity, barefoot working on lands, and adaptation of sensory inputs to stones, sand granules, and thorns to which the agricultural population is most often exposed. Thus, the pinprick sensation over a 10g monofilament pressure in these populations could have been not detected because of their adaptive sensory feedback. Overall, we found that 10 g monofilament was not only inaccurate in determining the DPN among the Indian population, but the stratification in our study also enhanced the inaccuracy. Based on the result of hypothesis testing using the χ2 Test, all four hypotheses got rejected at the level of significance of 0.05. We found that the accuracy of prediction using 10g monofilament is dependent on the use of footwear in general, the use of slippers at home, and occupation such as agriculture among the Indian population. Further research is to be done in determining the current monofilament which has higher sensitivity and sensitivity for the Indian diabetic population. It is also suggested that the above stated factors could have influenced not only the 10 g monofilament, but a difference could be observed in the VPT also. This was evident from findings of our study in 12 out of 120 cases, where the VPT value were less than 20 V, but they were having symptoms of neuropathy on MNSI. These findings further necessitate to redefine the vibration threshold among Indian population.

## Conclusion

The 10g monofilament is a prevailing instrument used in screening the diabetic foot for loss of protective sensation. A properly calibrated device must be used to ensure that 10g of linear pressure is being applied so that a true measurement is being assessed. This study concludes that the accuracy of 10 g monofilament for detecting DPN among the Indian population is limited. The accuracy further diminishes with variations in occupation, socioeconomic and religious practice. The associated factors affecting the accuracy of 10 g monofilament could have affected the VPT findings also which underscores that reliance solely on these factors or the 10g monofilament test alone is not sufficient for diagnosing DPN. Further investigations are required to determine the correct monofilament in clinical use for accurately detecting DPN among the Indian diabetic population.

## Supporting information

S1 Data (XLSX)
